# Oral Microbiome in Patients with Oesophageal Squamous Cell Carcinoma

**DOI:** 10.1038/s41598-019-55667-w

**Published:** 2019-12-13

**Authors:** Qian Wang, Yuting Rao, Xiaobing Guo, Na Liu, Shuxiu Liu, Peipei Wen, Shuang Li, Yuan Li

**Affiliations:** Department of Laboratory Medicine, the First Affiliated Hospital of Zhengzhou University, Zhengzhou, China

**Keywords:** Bacteria, Microbial communities

## Abstract

To investigate the oral microflora of patients with oesophageal squamous cell carcinoma (ESCC), saliva samples were collected from 20 patients with ESCC and 21 healthy controls. The V3-V4 region of 16S rDNA was amplified and sequenced by the Illumina MiSeq high-throughput sequencing platform. The final sequences were used for OTU analysis. Alpha and beta diversity analysis showed that the bacterial diversity and richness of the ESCC group were lower than those of the control group, while the variability of the ESCC group was higher than that of the control group. According to the Metastats difference analysis and LEfSe analysis, the high risk of ESCC may be related to *Actinomyces* and *Atopobium*, while the healthy control group is closely related to *Fusobacterium* and *Porphyromonas* (the analysis was performed at the genus level). The establishment of the relationship between oral microbiota and risk of ESCC may lead to significant advances in understanding the aetiology of cancer and may open a new research paradigm for cancer prevention.

## Introduction

Oesophageal cancer (EC) is the eighth most common cancer and the sixth most common cause of cancer-related death worldwide^1^. The 5-year survival rate of this cancer is approximately 13–18% due to a lack of accurate early screening methods and efficient treatment^2^. There are two major histological types of oesophageal cancer: oesophageal squamous cell carcinoma (ESCC) and oesophageal adenocarcinoma (EAC). EAC is the most common type in developed countries, while ESCC is dominant in developing countries^3,4^. It is worth noting that more than 90% of the oesophageal cancer cases in China are ESCC^5^.

The risk factors for ESCC include drinking, smoking, diet, chemical factors, infections, family history of oesophageal cancer, genetic changes^6^, but its aetiology is still unclear. Recently, an association between indicators of poor oral hygiene and ESCC has attracted the attention of researchers. A case-control study in Kashmir reported an increased risk of ESCC in those with poor oral hygiene^7^. A previous investigation showed that tooth loss and reduced tooth brushing can increase the risk of ESCC^8^. Furthermore, poor oral health was represented as a risk factor for oesophageal squamous dysplasia, a precursor lesion of ESCC^9^. The human oral cavity is colonized by more than 700 different bacterial species, that is, oral microbiome^10^. Current studies have shown that the balance of oral microbiology is essential to maintaining oral health. Poor oral health can be seen as the imbalance of the oral microbiome^11^.

However, the oral microbiome definitely plays a role in dental caries and periodontal disease. It may play a role in the other conditions (diabetes^12^, cancer^13,14^ etc). *Helicobacter pylori* was first diagnosed in 1994 as an infection associated with human cancer by the International Agency for Research on Cancer^15^. Evidence suggests that the flora is a driver of tumourigenesis. The human intestinal microbiota has been hypothesised to promote the formation of colorectal cancer^16,17^. The levels of *Enterococcus faecalis* in the faecal flora of patients with colorectal cancer are significantly higher than those in the faecal flora of polyp patients and healthy people, which may mean that bacteria may induce colorectal cancer^18^. Furthermore, although it is not known how *M. pneumoniae* infection induces the progression of lung cancer, the relationship between *M. pneumoniae* infection and lung cancer is biologically plausible^19^. Therefore, the relationship between the researched flora and tumour development is of great significance for tumour prevention and early treatment.

Streptococci are the dominant microorganisms found in the oesophagus. However, the conversion of Gram-positive bacteria to Gram-negative bacteria in oesophagitis and Barrett’s oesophagus may be related to the pathogenesis of oesophageal cancer^20^. There were many differences in the microbial composition between Barrett’s oesophagus patients and the control group. In the case–control study of patients with and without Barrett’s oesophagus from Snider *et al*., gene sequencing of 16s rRNA microorganisms was performed and further verified by qPCR. The results showed that the relative abundance of *Firmicutes* was significantly increased, and the relative abundance of *Proteobacteria* was significantly reduced in BE (Barrett’s oesophagus) patients^21^. BE, a chronic inflammatory disease associated with cancer progression, is a risk factor for oesophageal cancer^22^. Therefore, we suspect that there is also a difference in the oral flora of patients with oesophageal cancer and normal people. In addition, two studies have been conducted on oral flora related to ESCC. It is worth noting that Chen *et al*.^23^ and Peters *et al*.^24^ both used 16S rDNA analysis, OTU clustering, bioinformatics analysis and statistical analysis, while Peters *et al*.^24^ also analysed the association between EAC and oral flora; both studies found that the richness of *Porphyromonas gingivalis* leads to a higher risk of ESCC, but Peters *et al*.^24^ did not observe a significant correlation between overall microbial diversity or composition and the risk of EAC or ESCC. While Chen *et al*.^23^ showed that ESCC subjects had an overall decreased microbial diversity compared to control subjects (P < 0.001), patients with ESCC had decreased levels of the genera *Lautropia*, *Bulleidia*, *Catonella*, *Corynebacterium*, *Moryella*, *Peptococcus* and *Cardiobacterium* compared to non-ESCC subjects. However, Chen *et al*.^23^ did not extract DNA using bead-beating to disrupt the cells, which might affect the composition and diversity of the oral microbiota. We do not know whether geographical differences or differences in research methods caused the different research results. However, there is very little research on this group of Han people in China; therefore, it was necessary to carry out the research in this article^23,24^. In the current study, we aimed to investigate the potential association between oral microbiota in saliva and the risk of ESCC using the 16S rDNA amplicon sequencing approach based on a case-control study conducted in Henan with a high incidence of ESCC.

## Results

### Patient samples

A total of 20 ESCC patients (ESCC group, 14 men and 6 women) and 21 healthy controls (control group, 12 men and 9 women) were included in the study. Statistical analysis showed no significant differences in age, gender, smoking, education, alcohol consumption, BMI, vegetable and fruit intake and daily brushing frequency between the two groups. (P > 0.05) (Table 1).

**Table 1:** Demographic characteristics of subjects with oesophageal squamous cell carcinoma (ESCC) and healthy controls (control).

		Control (%) n = 21	ESCC (%) n = 20	P
Age (years, mean ± SD)		65.14 ± 6.12	65.90 ± 8.73	0.748^a^
Sex	Men	12 (57.14)	14 (70.00)	0.393^b^
	Women	9 (42.86)	6 (30.00)	
BMI (kg/m^2^)	<25	14 (66.7)	17 (85.0)	0.172^b^
	≥25	7 (33.3)	3 (15.0)	
Education	Illiterate	6 (28.6)	5 (25.0)	0.796^b^
	Literate	15 (71.4)	15 (75.0)	
Smoking	Never	11 (52.4)	7 (35.0)	0.262^b^
	Ever	10 (47.6)	13 (65.0)	
Alcohol drinking	Never	12 (57.1)	11 (55)	0.890^b^
	Ever	9 (42.9)	9 (45)	
Fruit and vegetable intake	<25 g	7 (33.3)	9 (45.0)	0.444^b^
	≥25 g	14 (66.7)	11 (55.0)	
Daily tooth brushing frequency	<2	12 (57.1)	15 (75.0)	0.228^b^
	≥2	9 (42.9)	5 (25.0)	

^a^Differences were detected using the independent sample T test.

^b^Differences were detected using the chi-square test.

Twenty patients with ESCC (ESCC group, 14 males and 6 females) and 21 control subjects (control group, 12 males and 9 females) were enrolled in the study.

### Sequencing data

After high-throughput sequencing, a total of 2,443,205 16S rDNA gene reads were obtained, and after chimeric sequences were removed, 2,173,904 effective sequences were obtained and used for further analysis. The average read length was 457 bp. (Supplementary Fig. S1 and Table S1).

### Relative abundance of species

**Figure 1:**
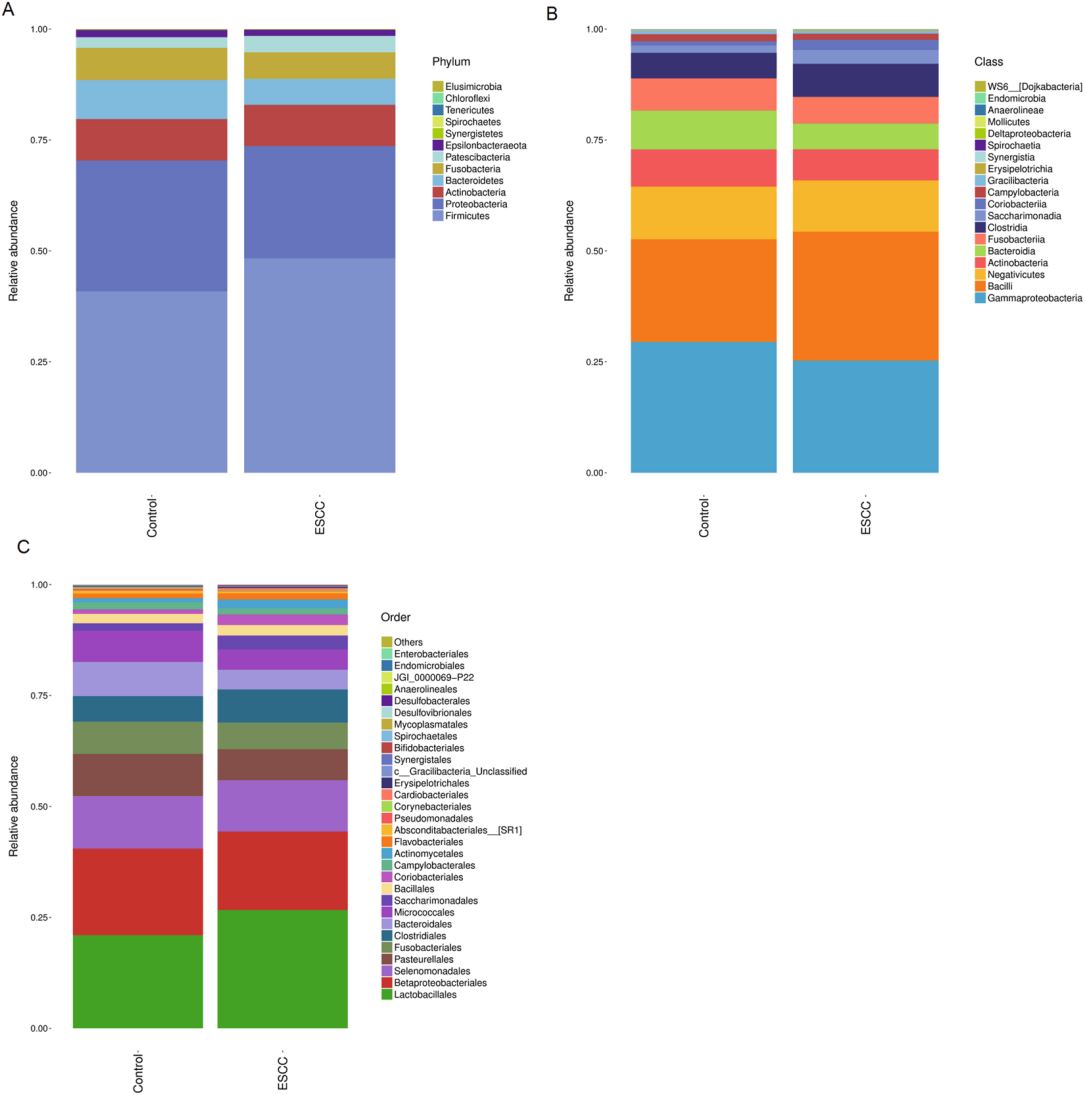
(**A**–**C**) are the heatmaps of the two groups of bacteria at the phylum, class and order levels, respectively. ESCC: oesophageal squamous cell carcinoma.

At the phylum level, the ESCC group had a higher proportion of *Firmicutes* than the healthy group. At the class level, the ESCC group had a lower proportion of *Gammaproteobacteria* and a higher proportion of *Bacilli* than the healthy group. At the order level, the ESCC group had a higher proportion of *Lactobacillales* (Fig. 1).

### Alpha diversity analysis

**Figure 2:**
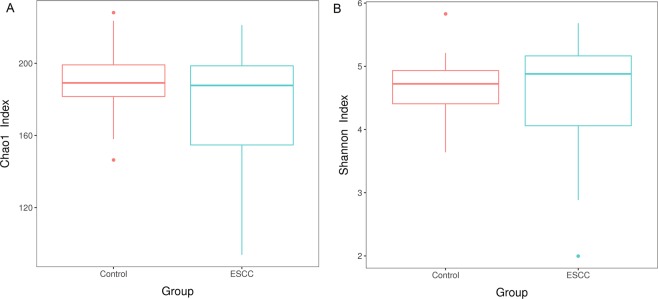
The alpha diversity of oral bacterial communities was analysed and compared among the ESCC and healthy control groups. The box plots are constructed based on the alpha diversity index table, and the difference between the alpha diversity index groups is analysed. Each box plot shows the minimum, the first quartile, the median, the third quartile and maximum values of the sample within the group. ESCC: oesophageal squamous cell carcinoma.

Good’s coverage was 99.9% or 100% for sequences in the ESCC samples and control samples (Supplementary Table S2), indicating that the sequences measured in each sample represented almost all the bacterial sequences in the sample. From the comparisons of the community diversity indices (Shannon and Simpson index) and richness indices (ACE and Chao) (Fig. 2 and Table 2), it was found that the ESCC group displayed slightly lower diversity and richness than the control groups based on the mean, but there was no significant difference between the two groups (P > 0.05) (Table 2).

**Table 2:** Alpha diversity analysis and statistical analysis.

	ESCC	Control	P
Ace	180.08	187.58	0.346^a^
Chao1	179.07	189.44	0.219^a^
Shannon	4.55	4.66	0.624^b^
Simpson	0.88	0.92	0.478^c^

The first two columns are the mean values of the two groups in these four indices.

^a^Independent sample T test.

^b^Correction Student’s t test.

^c^Wilcoxon rank-sum test.

### Beta diversity analysis

**Figure 3:**
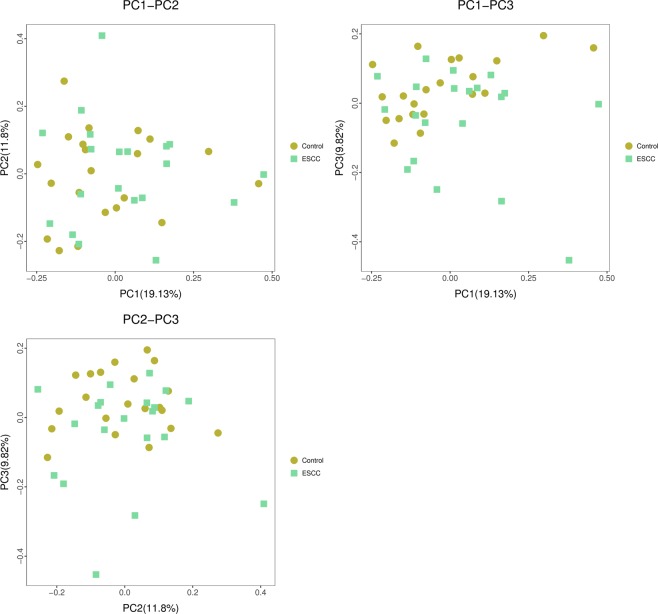
PCoA analysis; samples in the same group are represented by the same colour and shape. The percentage after the main coordinate represents the contribution to the sample difference. The distance of the sample point represents the similarity of the microbial community in the sample. The closer the distance is, the higher the similarity. ESCC: oesophageal squamous cell carcinoma.

**Figure 4:**
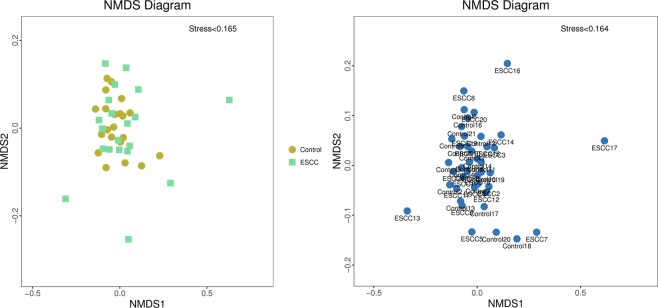
NMDS analysis (non-metric multi-dimensional scale) analysis; the left figure is analysed by group, and the right figure is analysed by each sample. ESCC: oesophageal squamous cell carcinoma.

Figure 3 shows the PCoA analysis (main coordinate analysis). The contributions of PC1, PC2 and PC3 for the sample differences were 19.13%, 11.8% and 9.82%, respectively. Statistical analysis results of the differences between the two groups are shown in Table 3 and Supplementary Table S3. PCoA analysis was based on the Bray-Curtis distance matrix for mapping analysis. As shown in Fig. 4, the stress < 0.2, indicating that NMDS can accurately reflect the degree of difference between samples. The variation in oesophageal bacteria in the ESCC group was greater than that in the control group. The NMDS analysis was based on the beta diversity distance matrix and was modelled using the R language vegan software package.

**Table 3:** Beta diversity analysis and statistical analysis.

PCoA	PCoA1	PCoA2	PCoA3
P	0.313^c^	0.815^a^	0.037^c^

^a^Independent sample T test.

^c^Wilcoxon rank-sum test.

### Metastats difference analysis

**Figure 5:**
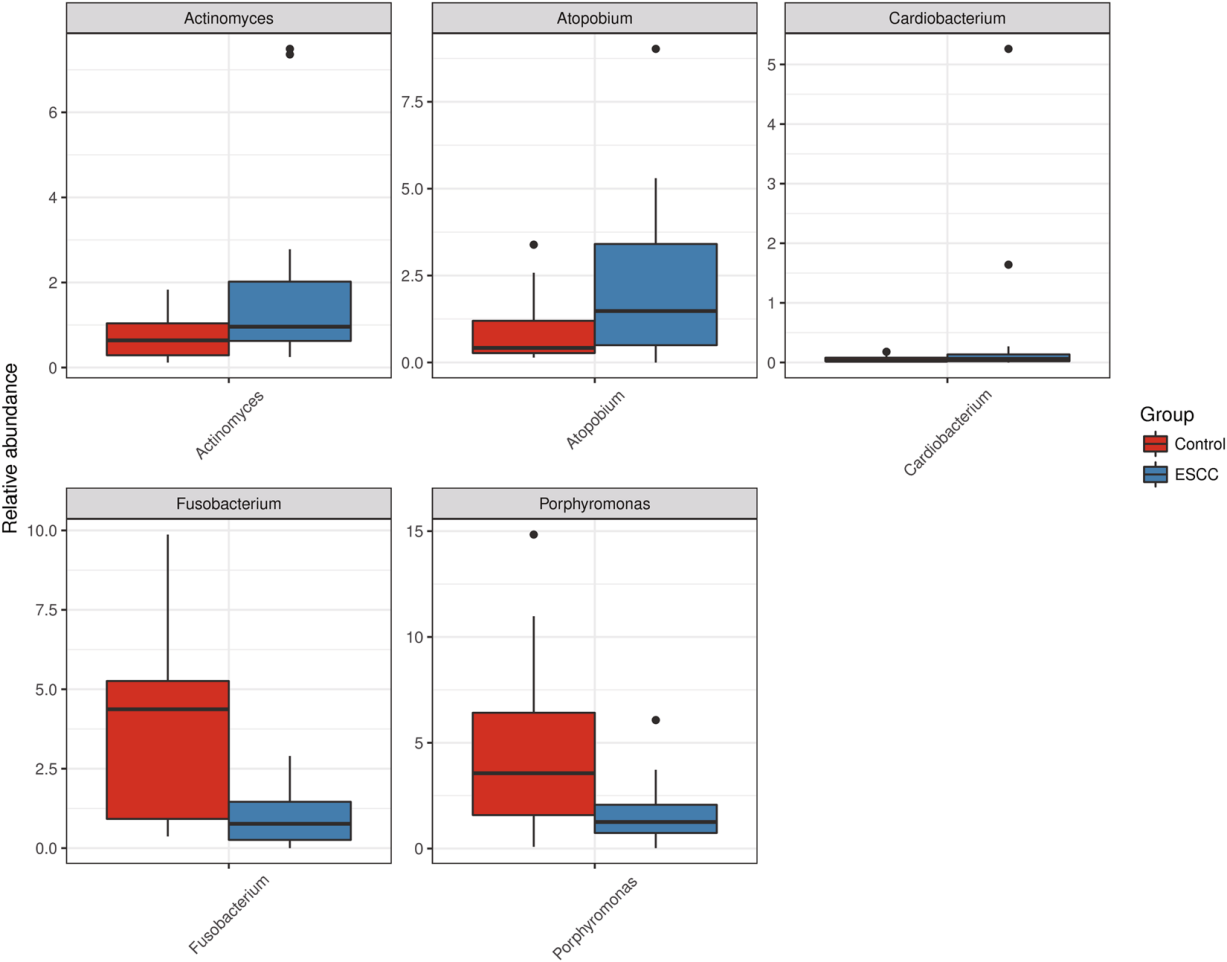
The abundance distribution of the five strains with the largest difference between the ESCC group and the control group is shown. The abscissa is the classification name of the five strains with the largest difference between the two groups, and the ordinate is the relative abundance of the strain. ESCC: oesophageal squamous cell carcinoma (the default is analysed at the genus level).

The top five strain differences between the two groups were *Actinomyces*, *Atopobium*, *Cardiobacterium*, *Fusobacterium* and *Porphyromonas*. The differences between the five bacterial genera in the two groups were significant (P < 0.05) (Fig. 5 and Supplementary Table S4) (the default was analysed at the genus level).

### LEfSe analysis

**Figure 6:**
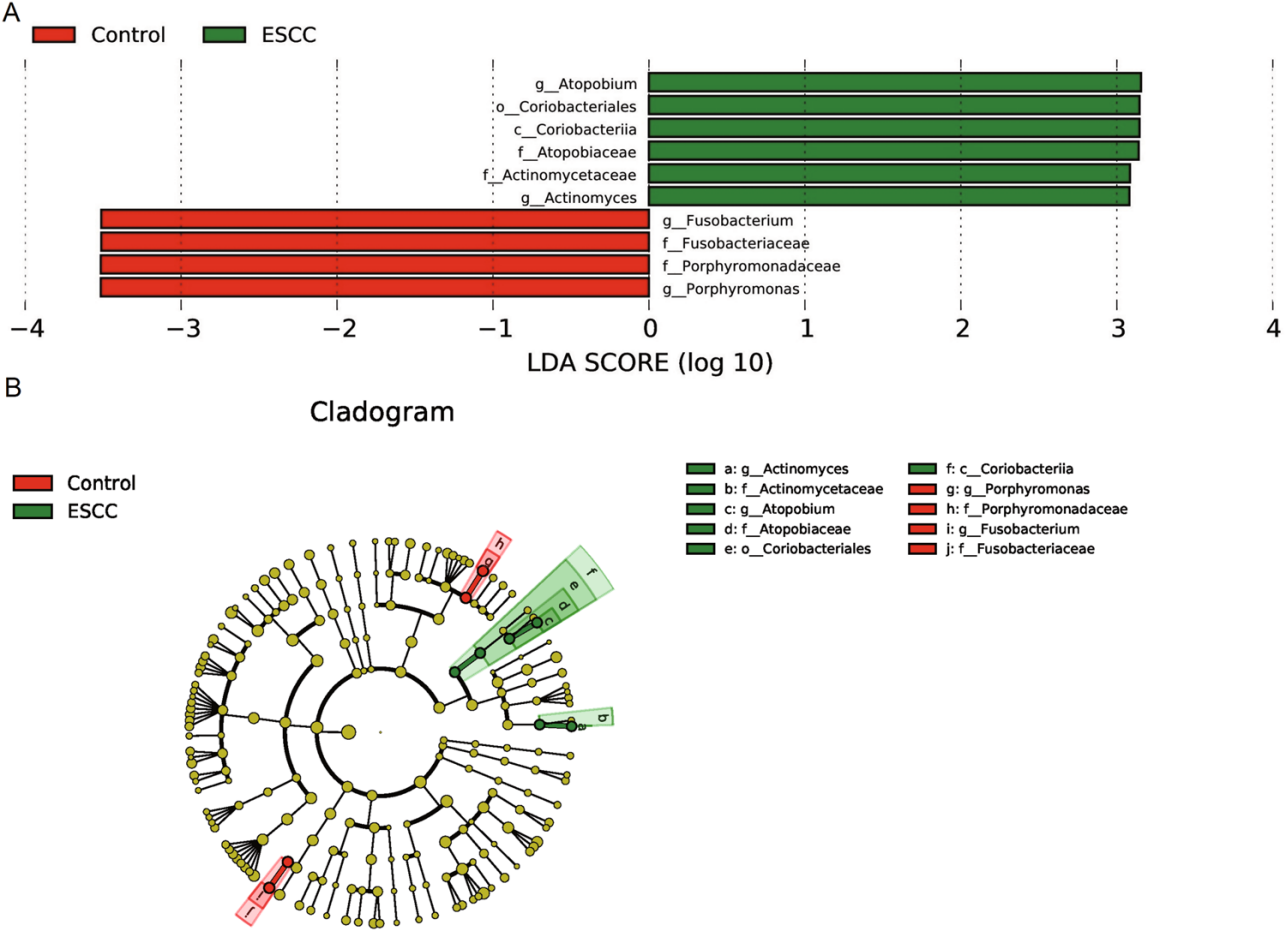
(**A**) The distribution bar chart of LDA values shows the species with LDA scores greater than the set value and the species with significantly different abundances in different groups. The length of the histogram represents the size of the impact of significantly different species. (**B**) The circle radiating from inside to outside represents the classification from the phylum to the genus level. Each small circle represents a classification at that level at different classification levels. The diameter of the small circle is proportional to the relative abundance. ESCC: oesophageal squamous cell carcinoma (c_ is class, o_ is order, f_ is family, and g_ is genus).

According to the LEfSe analysis, the specific bacteria related to the ESCC patient group were *Atopobium*, *Coriobacteriales*, *Coriobacteriia*, *Atopobiaceae*, *Actinomycetaceae* and *Actinomyces*. *Atopobium* and *Actinomyces* were identified at the genus level; *Coriobacteriales*, at the order level; *Coriobacteriia*, at the class level; and *Atopobiaceae* and *Actinomycetaceae*, at the family level. The specific bacteria of the healthy population were *Fusobacterium*, *Fusobacteriaceae*, *Porphyromonadaceae* and *Porphyromonas*. *Fusobacterium* and *Porphyromonas* were identified at the genus level, and *Fusobacteriaceae* and *Porphyromonadaceae* were identified at the family level (LDA score > 3) (Fig. 6A). In addition, the branch evolution relationship could be obtained through the cladogram in Fig. 6B.

## Discussion

China is a country with a high incidence of oesophageal cancer. Although the survival rate of early oesophageal cancer is still acceptable, once oesophageal cancer is found, it is usually in the late stage, and the survival rate at this stage is very low. Therefore, early detection, diagnosis and treatment are key to improving the prognosis of oesophageal cancer^6^. Risk factors such as gastroesophageal reflux disease, obesity, smoking and diet can no longer fully explain the increased incidence of oesophageal cancer, and upper gastrointestinal microflora may be another potential co-factor. The normal oesophagus was dominated by Streptococcus, while the oesophagitis and Barrett’s oesophagus were dominated by Gram-negative anaerobes^25^. Therefore, it is of great significance to study the correlation between the occurrence and development of oesophageal cancer and oral flora.

According to the alpha diversity analysis, it was found that the diversity and richness of the ESCC groups were slightly lower than those of the control groups, but the differences between the two groups were not significant. Chen *et al*.^23^ showed that patients with ESCC had low salivary microbial diversity compared to healthy controls. The overall microbial diversity of ESCC subjects decreased in this study. In 2014, Yu *et al*.^26^ believed that human oral microbial richness was negatively correlated with oesophageal squamous cell dysplasia. A study in China showed that a decreased microbial richness in the upper digestive tract was associated with cancer-predisposing conditions of the stomach and oesophagus. They believe that individuals with low oesophageal microbial complexity are more prone to oesophageal squamous dysplasia, while oesophageal squamous epithelial dysplasia is a prerequisite for oesophageal cancer, which is consistent with our results^26^. NMDS analysis showed that the bacterial variation in the ESCC group was greater than that in the control group. Therefore, it can be speculated that patients with ESCC may correspond to microflora with low diversity and high variability. However, it is not known whether the disease leads to a decrease in the microflora diversity and richness or whether low bacterial diversity and richness induce the diseases.

From the relative abundance of species analysis, it was found that the ESCC group had a higher proportion of *Firmicutes*, *Bacillus*, *Lactobacillus* and a lower proportion of *Gammaproteobacteria* than the control group. From Liu *et al*.^27^, compared to the healthy control group, *Firmicutes* in the ESCC group showed a relatively high abundance, while *Proteobacteria* showed a lower relative abundance, which is consistent with our analysis. According to the Metastats difference analysis and LEfSe analysis, the high risk of ESCC may be related to *Actinomyces* and *Atopobium*, while the healthy control group is closely related to *Fusobacterium* and *Porphyromonas*. Another article compared the microbiota between patients with bladder cancer and healthy controls and found that the abundance of *Actinomycetes* in patients with bladder cancer was higher than that in healthy people^28^. In 2019, Yachida *et al*.^29^ analysed the changes in the intestinal flora in patients with colorectal cancer; it was noted that *Actinomyces* and *Atopobium* were significantly increased in polypoid adenomas and intramucosal carcinomas. Increased abundance of *Actinomyces cardiffensis* was associated with a higher risk of EAC^24^. This indicates that *Actinomycetes* and *Atopobium* are associated with cancer.

It has been reported in a previous article that the oesophageal cancer group had a lower intake of fruits and vegetables and poor oral hygiene compared to the control group^30^. Our results were not the same. This may be because the number of cases was small, and the statistical results were not as accurate as those with larger samples; however, our results can reduce the influence of confounding factors such as smoking, drinking and tooth brushing. Other studies have shown that fruit and vegetable intake does not affect the risk of oesophageal cancer, which is consistent with our results^24^.

In this study, since ESCC subjects had periodontitis or gingivitis, to control the effects of confounding factors, we selected healthy controls with periodontitis or gingivitis. However, Chen *et al*.^23^ and Peters *et al*.^24^ indicated that the risk of ESCC is related to *P. gingivalis*, so the difference in the bioinformatics results obtained in this experiment may not be significant because patients with periodontitis or gingivitis themselves have a higher prevalence of ESCC than the normal population. Our study also has some limitations. Due to the lack of information on the periodontal status of the sample, the periodontal condition and the severity of the periodontal disease are not elaborated. It is impossible to determine whether the periodontal pathogen is not related to periodontal disease.

In summary, the oral microflora of patients with ESCC and a healthy control group were compared and analysed in our study. According to the alpha and beta diversity analysis, it was found that patients with ESCC may correspond to microflora with low diversity and high variability. According to the Metastats difference analysis and LEfSe analysis, the high risk of ESCC may be related to *Actinomyces* and *Atopobium*, while the healthy control group is closely related to *Fusobacterium* and *Porphyromonas* (the analysis was performed at the genus level). The establishment of the relationship between oral microbiota and risk of ESCC may lead to significant advances in understanding the aetiology of cancer and may open a new research paradigm for cancer prevention.

## Conclusions

According to the alpha and beta diversity analysis, compared with healthy control groups, ESCC has lower bacterial abundance and diversity and greater variability. According to the Metastats difference analysis and LEfSe analysis, the high risk of ESCC may be related to *Actinomyces* and *Atopobium*, while the healthy control group is closely related to *Fusobacterium* and *Porphyromonas* (the analysis was performed at the genus level).

## Methods

### Participants

All subjects in the study were of Han nationality, native Henan and local residents, who had lived in Henan for at least 5 years before sampling. Subjects who met the following criteria were excluded from the study: having any oral mucosal lesions; having bacterial or viral infections in tonsil, salivary glands or throat within 1 month before sampling; receiving any periodontal treatment within 6 months; receiving antibiotics or non-steroidal anti-inflammatory drugs in the previous 1 month; having invasive surgery, radiotherapy and chemotherapy in the past year; being in menstruation, gestation or lactation or taking oral contraceptives. Furthermore, patients with ESCC were clearly diagnosed by electronic gastroscopy and histopathology, with no related surgery, radiotherapy or chemotherapy for oesophageal cancer before sampling. Patients with other tumour histories, chronic diseases such as hypertension, diabetes and heart disease, and a history of infectious diseases were excluded. Healthy individuals were defined as subjects with no tumour history, chronic history of hypertension, diabetes and heart disease, or infectious diseases.

ESCC patients were recruited mainly from the Department of Thoracic Surgery at the First Affiliated Hospital of Zhengzhou University and Henan Cancer Hospital during the period from September 2017 to February 2018 (n = 20). Controls were those who were recruited during November of 2017 and February of 2018 (n = 21).

### Information collection

In this study, a questionnaire that was conducted face-to-face with all subjects by trained interviewers was designed to obtain comprehensive information about the subjects. The questionnaire included information about age, sex, race, education, body mass index (BMI), religious faith, smoking status, alcohol consumption, fruit and vegetable intake, times of tooth brushing per day and exclusion criteria. Professional dentists determined the periodontal status of subjects. All subjects had gingivitis or periodontitis.

### Saliva sample collection

All subjects were asked to have an empty stomach and not perform any oral hygiene procedure on the morning of the sampling. Saliva samples were collected from all subjects between 6:30 and 8:00 am. The participants were instructed to wash their mouth with pure water prior to sampling, followed by collection of at least 5 ml unstimulated saliva in a sterile cup. The saliva was then divided into 2 ml sterile EP tubes. Finally, the samples were kept frozen at −80 °C until use.

### Sample processing

DNA was extracted from saliva samples using the QIAamp DNA Microbiome Kit (Qiagen, Hilden, Germany) per the manufacturer’s recommendations. The QIAamp DNA Microbiome Kit can effectively deplete host DNA and fully extract DNA from bacteria, including Gram-positive bacteria. DNA concentration and purity were estimated by the A260/A280 and A260/A230 ratios using a Nano Drop 2000 Spectrophotometer (Thermo Fisher Scientific), and molecular degradation was assessed by agarose gel electrophoresis. The V3 and V4 hypervariable regions of bacteria and archaea 16S rDNA were amplified using forward primers containing the sequence “CCTACGGRRBGCASCAGKVRVGAAT” and reverse primers containing the sequence “GGACTACNVGGGTWTCTAATCC”. At the same time, indexed adapters were added to the ends of the 16S rDNA amplicons. The library quality was detected by Agilent 2100 Bioanalyzer (Agilent Technologies, Palo Alto, CA, USA) and Qubit 2.0 Fluorometer (Invitrogen, Carlsbad, CA, USA). After the DNA library was mixed, 2 × 300 bp double-end sequencing (PE) was carried out according to the Illumina MiSeq (Illumina, San Diego, CA, USA) instrument instruction manual, and the sequence information was read by MiSeq Control Software (MCS).

### Sequence data analysis

The QIIME data analysis package was used for 16S rDNA data analysis. The forward and reverse reads were joined and assigned to samples based on barcodes and truncated by removing the barcode and primer sequence. Quality filtering on joined sequences was performed, and sequences that did not fulfil the following criteria were discarded: sequence length <200 bp, no ambiguous bases, mean quality score >=20. Then, the sequences were compared with the reference database (RDP Gold database) using the UCHIME algorithm to detect the sequences, and the chimeric sequences were removed. Finally, we obtained 2,173,904 high quality 16S rDNA gene sequence reads from 41 saliva samples. Effective sequences were grouped into operational taxonomic units (OTUs) using the clustering program VSEARCH 1.9.6 against the Silva 132 database pre-clustered at 97% sequence identity. The Ribosomal Database Program (RDP) classifier was used to assign taxonomic categories to all OTUs at a confidence threshold of 0.8.

### Quality control

All samples were processed by the same experimenter in the same experimental condition, and personnel were blinded to sample status. The separation and extraction of saliva were carried out in an aseptic laminar flow hood, and all steps were taken to ensure aseptic operation. Negative control samples (without DNA template) were used to detect possible reagent and environmental contamination in all sequencing batches. Furthermore, all samples were sequenced in the same batch.

### Statistical analysis and bioinformatics analysis

Using statistical methods (the independent sample T test and chi-square test), the differences in the age, education level, gender, BMI, smoking, alcohol consumption, fruit and vegetable intake and daily brushing frequency between the two groups were compared. Alpha diversity between groups was calculated using the independent sample T test, correction Student’s test and Wilcoxon rank-sum test. PCoA and NMDS analyses were used to estimate the similarity between samples. At the same time, Metastats difference analysis identified the species with different abundances difference at the genus level between groups. LEfSe analysis identified the differences between two groups of bacteria from all levels.

### Ethics approval

The study was approved by the institutional review board of the First Affiliated Hospital of Zhengzhou University and Henan Cancer Hospital. Prior to the study, all subjects provided written informed consent.

### Accordance

The methods were carried out in accordance with the relevant guidelines and regulations.

## Supplementary information

Supplementary Dataset 1

## Data Availability

All data generated or analysed during this study are included in this published article (and its Supplementary Information Files). The data has been uploaded to NCBI. SRA accession: PRJNA587078.
