# Supplementary material for: Oral Microbiome in Patients with Oesophageal Squamous Cell Carcinoma
Source: Sci Rep. 2019 Dec 13;9:19055. doi: 10.1038/s41598-019-55667-w (PMC6910992; doi:10.1038/s41598-019-55667-w)
Supplement: Supplementary file 1 — Supplementary Dataset 1 [file 41598_2019_55667_MOESM1_ESM.pdf]

Oral Microbiome in Patients with Oesophageal Squamous Cell Carcinoma

Qian Wang†, Yuting Rao†, Xiaobing Guo*, Na Liu, Shuxiu Liu, Peipei Wen, Shuang Li, Yuan Li

Department of Laboratory Medicine, the First Affiliated Hospital of Zhengzhou University, Zhengzhou, China

†Contributed equally to the work

***CORRESPONDING AUTHOR**

Xiaobing Guo

E-mail: gxbing928@zzu.edu.cn

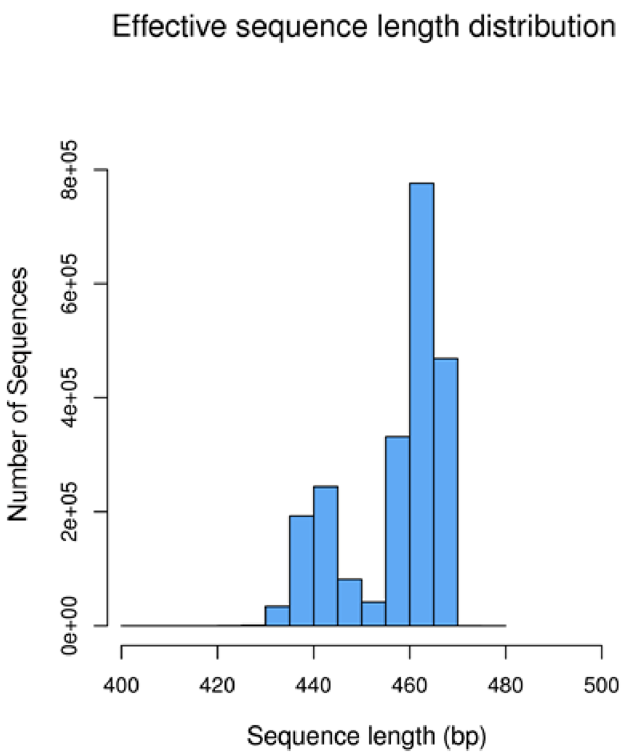

Supplementary Fig. S1. After optimizing the sequencing raw data, effective sequence length distribution statistics were obtained. The abscissa is the sequence length (bp), and the ordinate is the sequence number.

Supplementary Table S1. Sequencing data

Sample	#PE reads	#Nochimaera	AvgLen (bp)	GC (%)
**Control10**	56725	50328	454.21	52.54
**Control11**	65787	58060	457.46	52.5
**Control12**	70287	62686	457.48	52.33
**Control13**	68421	57795	459.47	52.37
**Control14**	75225	65128	457.18	52.76
**Control15**	67889	57478	456.08	52.18
**Control16**	86015	75677	456.83	52.44
**Control17**	64035	58547	457.4	53.11
**Control18**	56573	45935	457.18	53.37
**Control19**	61671	55042	456.23	53.25
**Control1**	48398	45289	458.89	52.26
**Control20**	51519	46643	457.95	53.49
**Control21**	55466	51092	458.82	51.95
**Control2**	51623	46686	457.08	52.51
**Control3**	50354	44843	458.32	52.51
**Control4**	49518	44719	455.88	51.97
**Control5**	58924	53904	459.54	52.17
**Control6**	59177	55054	457.59	52.13
**Control7**	50973	45338	457.98	52.94
**Control8**	67001	60825	458.07	52.77
**Control9**	67007	61045	456.59	51.93
**ESCC10**	54363	49872	456.43	52.63
**ESCC11**	55655	51507	459.7	52.71
**ESCC12**	63178	57329	457.55	53
**ESCC13**	59349	56292	458.55	51.83
**ESCC14**	53250	44208	455.97	52.98
**ESCC15**	62701	55176	455.6	52.86
**ESCC16**	51728	48206	454.18	53
**ESCC17**	76104	70487	461.73	53.78
**ESCC18**	73663	64587	456.74	52.77
**ESCC19**	54864	50196	459.53	52.45
**ESCC1**	67686	57907	454.47	52.63
**ESCC20**	69151	63068	455.92	52.45
**ESCC2**	50099	41905	456.96	52.94
**ESCC3**	48471	38519	454.54	52.48
**ESCC4**	51918	43161	457.4	53.24

**ESCC5**	50249	42021	458.83	52.82
**ESCC6**	50709	45297	460.23	52.48
**ESCC7**	50119	45157	456.82	53.73
**ESCC8**	50875	44230	457.09	52.41
**ESCC9**	66485	62665	458.21	52.84

Supplementary Table S2. Alpha diversity analysis

Sample	Ace	Chao1	Shannon	Simpson	Goods_coverage
Control1	193.723	189.12	4.407	0.9	1
Control2	209.779	206	4.731	0.911	0.999
Control3	187.843	186.059	4.61	0.911	0.999
Control4	201.349	212.833	4.933	0.941	0.999
Control5	177.833	183	3.987	0.876	0.999
Control6	180.511	176.2	4.464	0.875	1
Control7	182.08	190.273	4.771	0.937	0.999
Control8	221.369	228.083	4.833	0.921	0.999
Control9	199.577	199.125	5.829	0.965	1
Control10	183.806	185.545	5.087	0.943	1
Control11	196.575	194.875	5.211	0.952	0.999
Control12	193.857	195.071	4.925	0.936	0.999
Control13	150.072	146.4	4.061	0.898	0.999
Control14	176.997	181.6	5.021	0.943	1
Control15	188.193	183.714	5.077	0.948	1
Control16	205.785	223.545	4.016	0.844	0.999
Control17	174.506	176.615	4.722	0.932	0.999
Control18	157.692	158	4.379	0.919	0.999
Control19	164.074	161.882	4.708	0.934	1
Control20	194.734	196	3.64	0.861	0.999
Control21	198.772	204.2	4.523	0.916	0.999
ESCC1	198.092	193.667	5.457	0.961	0.999
ESCC2	147.017	147.1	5.157	0.953	1
ESCC3	203.796	198.438	5.462	0.963	0.999
ESCC4	195.863	192.5	4.589	0.919	0.999
ESCC5	99.827	93.8	3.831	0.888	1
ESCC6	191.711	194.067	4.098	0.883	0.999
ESCC7	195.778	191.056	3.944	0.826	0.999
ESCC8	157.216	152.176	3.919	0.853	0.999
ESCC9	180.435	182.364	4.362	0.883	1
ESCC10	206.762	205.5	5.14	0.933	1
ESCC11	174.713	177.25	4.166	0.852	0.999
ESCC12	140.47	133.077	4.931	0.943	1
ESCC13	158.031	155.55	2.884	0.643	0.999
ESCC14	148.77	147.909	4.995	0.945	1
ESCC15	217.387	215.5	5.682	0.968	0.999
ESCC16	180.199	184.375	5.351	0.941	0.999

**ESCC17**	**163.614**	**180.111**	**1.997**	**0.466**	**0.999**
**ESCC18**	**202.916**	**199.056**	**4.978**	**0.936**	**0.999**
**ESCC19**	**223.431**	**221.2**	**4.829**	**0.91**	**0.999**
**ESCC20**	**215.606**	**216.769**	**5.189**	**0.921**	**0.999**

Supplementary Table S3. Beta diversity analysis. PCoA analysis

Sample	Axis.1	Axis.2	Axis.3
**Control1**	**-0.204**	**-0.028**	**-0.049**
**Control2**	**-0.162**	**0.274**	**-0.045**
**Control3**	**-0.115**	**-0.055**	**-0.002**
**Control4**	**-0.149**	**0.11**	**0.021**
**Control5**	**-0.247**	**0.027**	**0.112**
**Control6**	**-0.178**	**-0.227**	**-0.115**
**Control7**	**0.073**	**0.128**	**0.077**
**Control8**	**-0.083**	**0.136**	**-0.031**
**Control9**	**-0.095**	**0.071**	**-0.087**
**Control10**	**0.071**	**0.06**	**0.026**
**Control11**	**0.029**	**-0.071**	**0.131**
**Control12**	**-0.031**	**-0.114**	**0.059**
**Control13**	**-0.103**	**0.087**	**0.164**
**Control14**	**0.004**	**-0.101**	**0.126**
**Control15**	**-0.077**	**0.009**	**0.039**
**Control16**	**-0.119**	**-0.215**	**-0.033**
**Control17**	**0.111**	**0.103**	**0.029**
**Control18**	**0.457**	**-0.029**	**0.16**
**Control19**	**0.149**	**-0.144**	**0.122**
**Control20**	**0.298**	**0.066**	**0.195**
**Control21**	**-0.216**	**-0.193**	**0.018**
**ESCC1**	**-0.079**	**0.117**	**-0.056**
**ESCC2**	**0.163**	**0.082**	**0.018**
**ESCC3**	**0.086**	**-0.071**	**0.044**
**ESCC4**	**0.013**	**0.065**	**0.043**
**ESCC5**	**0.01**	**-0.043**	**0.095**
**ESCC6**	**-0.231**	**0.121**	**0.078**
**ESCC7**	**0.473**	**-0.002**	**-0.003**
**ESCC8**	**-0.136**	**-0.18**	**-0.191**
**ESCC9**	**-0.077**	**0.073**	**0.128**
**ESCC10**	**-0.11**	**-0.06**	**-0.035**
**ESCC11**	**-0.108**	**0.188**	**0.047**
**ESCC12**	**0.174**	**0.087**	**0.029**
**ESCC13**	**-0.042**	**0.409**	**-0.249**
**ESCC14**	**0.13**	**-0.256**	**0.081**
**ESCC15**	**0.061**	**-0.078**	**0.035**
**ESCC16**	**0.164**	**0.03**	**-0.283**

**ESCC17**	**0.379**	**-0.085**	**-0.453**
**ESCC18**	**0.04**	**0.066**	**-0.059**
**ESCC19**	**-0.208**	**-0.147**	**-0.018**
**ESCC20**	**-0.116**	**-0.208**	**-0.167**

Supplementary Table S4. Metastats group difference analysis

Taxon	Group1_mean	Group1_variance	Group1_standard	Group2_mean	Group2_variance	Group2_standard	P_Value	Q_Value
Streptococcus	0.183471	0.006494	0.017585	0.197938	0.020262	0.03183	0.714286	1
Neisseria	0.185197	0.013481	0.025337	0.162937	0.015388	0.027738	0.55045	1
Veillonella	0.099958	0.002383	0.010653	0.087556	0.003191	0.012631	0.457542	1
Haemophilus	0.082472	0.00249	0.010889	0.063993	0.00613	0.017507	0.390609	1
Rothia	0.070489	0.00451	0.014655	0.045838	0.001233	0.007852	0.146853	1
Leptotrichia	0.032249	0.000458	0.004671	0.050582	0.002225	0.010548	0.125874	1
Porphyromonas	0.04506	0.00163	0.008811	0.016535	0.000226	0.003363	0.001998	0.540275
Fusobacterium	0.040198	0.001048	0.007065	0.00929	6.33E-05	0.001778	0.000999	0.540275
Granulicatella	0.021364	8.58E-05	0.002022	0.025828	0.000268	0.003662	0.288711	1
Gemella	0.021511	0.000207	0.003138	0.023907	0.000302	0.003887	0.616384	1
Abiotrophia	0.002219	1.64E-05	0.000883	0.039505	0.026081	0.036112	0.23976	1
Prevotella_7	0.021581	0.001156	0.00742	0.015325	0.000162	0.002848	0.5414	1

							59	
f__Saccharimonadaceae_Unclassified	0.012265	9.31E-05	0.002105	0.021123	0.000491	0.004953	0.102897	1
Peptostreptococcus	0.01395	0.000103	0.002217	0.017159	0.00023	0.003393	0.441558	1
Atopobium	0.008809	8.01E-05	0.001953	0.022282	0.000518	0.005091	0.012987	1
Campylobacter	0.016188	0.000114	0.002334	0.013934	0.000249	0.003527	0.593407	1
Actinomyces	0.007413	2.67E-05	0.001128	0.01755	0.00043	0.004639	0.023976	1
Capnocytophaga	0.009598	8.35E-05	0.001993	0.013535	0.000142	0.002666	0.246753	1
Parvimonas	0.010179	0.000164	0.002795	0.012424	0.000311	0.003942	0.676324	1
f__Neisseriaceae_Unclassified	0.00568	3.43E-05	0.001279	0.009825	0.0001	0.002236	0.154784	1
[Eubacterium]_nodatum_group	0.007185	2.97E-05	0.00119	0.007105	2.5E-05	0.001118	1	1
f__Ambiguous_taxa_Unclassified	0.006566	0.000164	0.002794	0.007674	0.00033	0.004062	0.85272	1
Aggregatibacter	0.009104	0.000133	0.002515	0.00463	4.53E-05	0.001505	0.088703	1
Oribacterium	0.005114	1.16E-05	0.000744	0.00868	7.23E-05	0.001901	0.255457	1
Megasphaera	0.00409	4.76E-05	0.001505	0.00767	9.3E-05	0.002157	0.219954	1
Dialister	0.003	1.06E-	0.00071	0.007	0.0001	0.00235	0.0	1

	547	05	1	419	11	4	86405	
Prevotella	0.005198	0.000117	0.002356	0.00544	3.41E-05	0.001305	1	1
Selenomonas_3	0.003552	6.25E-06	0.000545	0.00678	0.000126	0.00251	0.125695	1
Unclassified_Unclassified	0.003509	1.07E-05	0.000714	0.0062	6.22E-05	0.001763	0.252969	1
Lachnoanaerobaculum	0.004233	7.3E-06	0.00059	0.00418	1.04E-05	0.00072	1	1
Ruminococcaceae_UCG-014	0.002814	1.29E-05	0.000784	0.004135	2.13E-05	0.001033	0.599129	1
Filifactor	0.002933	6.72E-06	0.000566	0.003445	1.66E-05	0.000911	0.785856	1
Stomatobaculum	0.003304	5.91E-06	0.000531	0.00279	1.73E-05	0.000931	1	1
f__P5D1-392_Unclassified	0.00249	1.29E-05	0.000785	0.003025	1.42E-05	0.000843	0.769305	1
Moraxella	0.003804	9.54E-05	0.002132	0.001535	2.12E-05	0.001029	0.227468	1
Lautropia	0.003094	7.38E-05	0.001875	0.002235	7.27E-06	0.000603	0.754339	1
Actinobacillus	0.003634	5.78E-05	0.001659	0.001619	3.53E-05	0.001328	0.227499	1
[Eubacterium]_brachy_group	0.002081	5.91E-06	0.000531	0.003125	4E-05	0.001414	0.539468	1
Corynebacterium	0.003171	0.000121	0.002404	0.00188	1.87E-05	0.000967	0.549648	1
Prevotella_6	0.002085	1.22E-05	0.000763	0.002775	1.77E-05	0.00094	0.5394	1

							68	
Cardiobacterium	0.000543	2.87E-07	0.000117	0.00405	0.000143	0.002677	0.018657	1
Shuttleworthia	0.000886	2.32E-06	0.000332	0.003625	4.52E-05	0.001503	0.101294	1
Candidatus_Saccharimonas	0.001905	2.66E-06	0.000356	0.00202	4.11E-06	0.000453	1	1
Centipeda	0.00199	9.06E-06	0.000657	0.001875	1.14E-05	0.000756	1	1
Mogibacterium	0.001709	2.05E-06	0.000312	0.00188	3.83E-06	0.000438	1	1
f__Lachnospiraceae_Unclassified	0.000238	2.36E-07	0.000106	0.00312	4.16E-05	0.001442	0.013422	1
Selenomonas	0.001852	5.57E-06	0.000515	0.00143	1.53E-06	0.000277	1	1
candidate_division_SR1_bacterium_taxon_345	0.001405	2.86E-06	0.000369	0.001775	6.66E-06	0.000577	0.720179	1
Solobacterium	0.001114	8.33E-07	0.000199	0.00201	8.13E-06	0.000638	0.442101	1
f__Veillonellaceae_Unclassified	0.001381	4.62E-06	0.000469	0.001255	2.45E-06	0.00035	1	1
Johnsonella	0.001366	2.06E-06	0.000313	0.00091	9.23E-07	0.000215	1	1
Alloprevotella	0.001433	2.35E-05	0.001057	0.00075	1.36E-06	0.000261	1	1
Selenomonas_4	0.001085	2.01E-06	0.000309	0.00105	4.54E-06	0.000477	1	1
Tannerella	0.000785	5.12E-06	0.000494	0.0013	1.84E-06	0.000303	0.680029	1
Fretibacterium	0.001014	1.63E-06	0.000279	0.0009	1.7E-06	0.000292	1	1
Candidatus_Saccharibacteria_bacterium_UB2523	0.000609	1.12E-06	0.000231	0.000975	5.94E-06	0.000545	0.615984	1

**Anaeroglobus**	**0.001009**	**1.63E-06**	**0.000278**	**0.000435**	**2.51E-07**	**0.000112**	**1**	**1**
**Phocaeicola**	**0.000219**	**3.06E-07**	**0.000121**	**0.001215**	**1.51E-05**	**0.000868**	**0.237893**	**1**
**[Eubacterium]_saphenum_group**	**0.000719**	**1.71E-06**	**0.000286**	**0.00065**	**1.51E-06**	**0.000274**	**1**	**1**
**F0058**	**0.000543**	**2.2E-06**	**0.000324**	**0.000795**	**8.39E-07**	**0.000205**	**0.615984**	**1**
**Eikenella**	**0.000576**	**5.33E-07**	**0.000159**	**0.000755**	**2.01E-06**	**0.000317**	**0.616073**	**1**
**Bifidobacterium**	**0.000148**	**9.26E-08**	**6.64E-05**	**0.001**	**1.46E-05**	**0.000854**	**0.237893**	**1**
**Comamonas**	**0.000129**	**4.71E-08**	**4.74E-05**	**0.000925**	**7.79E-06**	**0.000624**	**0.237893**	**1**
**Bergeyella**	**0.00049**	**6.91E-07**	**0.000181**	**0.0005**	**7.41E-07**	**0.000192**	**1**	**1**
**Olsenella**	**0.000476**	**3.09E-06**	**0.000384**	**0.000285**	**1.7E-07**	**9.21E-05**	**1**	**1**
**Lactobacillus**	**0.000276**	**1.28E-06**	**0.000247**	**0.0004**	**5.63E-07**	**0.000168**	**1**	**1**
**Treponema_2**	**0.000405**	**2.09E-06**	**0.000315**	**0.00019**	**3.15E-08**	**3.97E-05**	**1**	**1**
**Chryseobacterium**	**0.000557**	**6.51E-06**	**0.000557**	**0**	**0**	**0**	**1**	**1**
**Cryptobacterium**	**0.000181**	**1.85E-07**	**9.38E-05**	**0.00039**	**2.54E-07**	**0.000113**	**0.487805**	**1**
**Kingella**	**0.000405**	**7.57E-07**	**0.00019**	**7.5E-05**	**2.51E-08**	**3.55E-05**	**1**	**1**
**Erysipelotrichaceae_UCG-006**	**0.000224**	**9.49E-08**	**6.72E-05**	**0.000265**	**3.45E-07**	**0.000131**	**0.48793**	**1**
**Catonella**	**0.000267**	**2.7E-07**	**0.000113**	**0.00019**	**4.62E-08**	**4.81E-05**	**1**	**1**
**Alloscardovia**	**0.000**	**7.19E-**	**0.00018**	**0.000**	**3.46E-**	**0.00013**	**1**	**1**

	214	07	5	235	07	1		
**f__Family_XIII_Unclassified**	**0.000281**	**5.31E-07**	**0.000159**	**0.00016**	**7.52E-08**	**6.13E-05**	**1**	**1**
**Butyrivibrio_2**	**7.14E-05**	**3.41E-08**	**4.03E-05**	**0.000375**	**3.81E-07**	**0.000138**	**0.487805**	**1**
**Bacteroides**	**9.04E-05**	**4.78E-08**	**4.77E-05**	**0.000295**	**1.66E-07**	**9.1E-05**	**0.487805**	**1**
**Scardovia**	**0.00011**	**4.19E-08**	**4.47E-05**	**0.000265**	**1.69E-07**	**9.18E-05**	**0.48793**	**1**
**Faucicola**	**0.000271**	**1.44E-06**	**0.000262**	**6E-05**	**3.3E-08**	**4.06E-05**	**1**	**1**
**Mycoplasma**	**0.000314**	**2.07E-06**	**0.000314**	**5E-06**	**5E-10**	**5E-06**	**1**	**1**
**Pyramidobacter**	**0.000148**	**2.7E-07**	**0.000113**	**0.00014**	**1.78E-07**	**9.44E-05**	**1**	**1**
**Desulfovibrio**	**7.62E-05**	**5.79E-08**	**5.25E-05**	**0.000175**	**1.16E-07**	**7.6E-05**	**1**	**1**
**f__Corynebacteriaceae_Unclassified**	**0.00011**	**2.09E-08**	**3.15E-05**	**0.000115**	**7.93E-08**	**6.3E-05**	**1**	**1**
**Peptococcus**	**2.38E-05**	**4.9E-09**	**1.53E-05**	**0.000195**	**2.73E-07**	**0.000117**	**1**	**1**
**Desulfobulbus**	**7.14E-05**	**1.11E-08**	**2.3E-05**	**0.000145**	**8.16E-08**	**6.39E-05**	**1**	**1**
**Bulleidia**	**5.71E-05**	**6.57E-09**	**1.77E-05**	**0.00015**	**4.68E-08**	**4.84E-05**	**1**	**1**
**Mobiluncus**	**2.86E-05**	**5.14E-09**	**1.56E-05**	**0.000165**	**2.07E-07**	**0.000102**	**1**	**1**
**Pseudoramibacter**	**1.9E-05**	**1.62E-09**	**8.78E-06**	**0.000165**	**3.59E-07**	**0.000134**	**1**	**1**
**Propionivibrio**	**4.76E-06**	**4.76E-10**	**4.76E-06**	**0.000165**	**4.48E-07**	**0.00015**	**1**	**1**
**Flexilinea**	**2.86E-05**	**2.14E-09**	**1.01E-05**	**0.00015**	**1.43E-07**	**8.45E-05**	**1**	**1**
**Parascardovia**	**8.09E-05**	**6.86E-08**	**5.72E-05**	**6E-05**	**1.62E-08**	**2.85E-05**	**1**	**1**
**Erysipelotrichaceae_UCG-004**	**0**	**0**	**0**	**0.00014**	**3.64E-07**	**0.000135**	**1**	**1**

**[Eubacterium]_coprostanoligenes_group**	**3.33E-05**	**4.33E-09**	**1.44E-05**	**9E-05**	**5.57E-08**	**5.28E-05**	**1**	**1**
**Candidatus_Endomicrobium**	**0.000119**	**9.06E-08**	**6.57E-05**	**1E-05**	**9.47E-10**	**6.88E-06**	**1**	**1**
**W5053**	**6.67E-05**	**6.33E-09**	**1.74E-05**	**6E-05**	**1.62E-08**	**2.85E-05**	**1**	**1**
**Prevotella_2**	**7.62E-05**	**5.68E-08**	**5.2E-05**	**3E-05**	**3.26E-09**	**1.28E-05**	**1**	**1**
**Pelospora**	**2.38E-05**	**2.9E-09**	**1.18E-05**	**7.5E-05**	**2.2E-08**	**3.31E-05**	**1**	**1**
**f__Atopobiaceae_Unclassified**	**6.67E-05**	**1.93E-08**	**3.03E-05**	**2.5E-05**	**1.97E-09**	**9.93E-06**	**1**	**1**
**Howardella**	**4.76E-05**	**4.62E-09**	**1.48E-05**	**6.5E-05**	**8.7E-09**	**2.09E-05**	**1**	**1**
**Rikenellaceae_RC9_gut_group**	**7.62E-05**	**7.98E-08**	**6.16E-05**	**1.5E-05**	**1.34E-09**	**8.19E-06**	**1**	**1**
**Lactococcus**	**6.66E-05**	**9.33E-08**	**6.66E-05**	**0**	**0**	**0**	**1**	**1**
**Escherichia-Shigella**	**1.9E-05**	**1.62E-09**	**8.78E-06**	**3E-05**	**8.53E-09**	**2.06E-05**	**1**	**1**
**Peptoanaerobacter**	**3.33E-05**	**4.33E-09**	**1.44E-05**	**1.5E-05**	**2.39E-09**	**1.09E-05**	**1**	**1**
**f__Enterobacteriaceae_Unclassified**	**3.33E-05**	**2.33E-08**	**3.33E-05**	**0**	**0**	**0**	**1**	**1**
**f__Lentimicrobiaceae_Unclassified**	**1.9E-05**	**4.61E-09**	**1.48E-05**	**1E-05**	**9.47E-10**	**6.88E-06**	**1**	**1**
**f__Pasteurellaceae_Unclassified**	**2.38E-05**	**3.91E-09**	**1.36E-05**	**0**	**0**	**0**	**1**	**1**
**Propionibacterium**	**0**	**0**	**0**	**1.5E-05**	**1.34E-09**	**8.19E-06**	**1**	**1**
